# The Human Virome in Health and Its Remodeling During HIV Infection and Antiretroviral Therapy: A Narrative Review

**DOI:** 10.3390/microorganisms14010050

**Published:** 2025-12-25

**Authors:** Federico Cesanelli, Irene Scarvaglieri, Maria Antonia De Francesco, Maria Alberti, Martina Salvi, Giorgio Tiecco, Francesco Castelli, Eugenia Quiros-Roldan

**Affiliations:** 1 Unit of Infectious and Tropical Diseases, Department of Clinical and Experimental Sciences, University of Brescia and ASST Spedali Civili di Brescia, 25123 Brescia, Italy; f.cesanelli@unibs.it (F.C.); i.scarvaglieri@unibs.it (I.S.); m.alberti035@studenti.unibs.it (M.A.); m.salvi026@unibs.it (M.S.); g.tiecco@unibs.it (G.T.); francesco.castelli@unibs.it (F.C.); 2 Highly Specialized Laboratory, ASST Spedali Civili of Brescia, 25123 Brescia, Italy; maria.defrancesco@unibs.it; 3 Institute of Microbiology, Department of Molecular and Translational Medicine, University of Brescia, 25123 Brescia, Italy

**Keywords:** human virome, HIV infection, antiretroviral therapy, microbiome, inflammation

## Abstract

The human virome represents a fundamental yet understudied component of the microbiome, influencing immune regulation and disease. Given the profound immune dysregulation and microbial imbalance associated with HIV infection, understanding virome alterations during HIV and antiretroviral therapy is essential. This narrative review seeks to integrate and discuss the latest evidence regarding the structure and behavior of the human virome in healthy individuals, in the context of HIV infection, and under antiretroviral therapy. A comprehensive literature search was performed in MEDLINE and Google Scholar for peer-reviewed English-language articles published up to November 2025. Studies describing virome composition, diversity, and interactions in people living with HIV, as well as antiretroviral-induced changes, were included. Reference lists of relevant papers were screened to identify additional sources. Data were extracted and synthesized narratively, emphasizing human studies and supported by evidence from primate models where applicable. HIV infection induces profound alterations in the human virome, notably an expansion of eukaryotic viruses such as *Anelloviridae*, *Adenoviridae*, and *Parvoviridae*, accompanied by reduced bacteriophage diversity. Antiretroviral therapy partially restores virome balance but fails to fully re-establish pre-infection diversity, with persistent enrichment of *Anelloviridae* reflecting incomplete immune reconstitution. Virome perturbations correlate with immune activation, microbial translocation, and inflammation, contributing to comorbidities despite virological suppression. Emerging evidence suggests regimen-specific effects, with integrase inhibitor-based therapies showing more favorable viromic recovery. HIV and antiretroviral therapy profoundly remodel the human virome, with lasting implications for immune homeostasis and chronic inflammation. The ongoing disruption of the virome highlights its promise as both a biomarker and a potential therapeutic target in the management of HIV. Longitudinal, multi-omic studies are needed to clarify the causal role of virome alterations and guide future interventions.

## 1. Introduction

The human body harbors a vast ecosystem of microorganisms, including bacteria, viruses, archaea, and fungi, which inhabit the gut, lungs, skin, and other organs, collectively forming the microbiome. Each body site hosts a unique microbial community—for instance, the gut, skin, or lung microbiome—which can be further divided into specific components such as the bacteriome or virome. Among these, the virome plays a particularly important role, representing the most diverse and abundant collection of parasitic entities in the body. It includes animal viruses, which may exist temporarily or persist chronically, as well as bacteriophages, which infect bacteria and can follow lytic or lysogenic cycles. Although research has extensively examined links between the bacteriome and major conditions like cancer, diabetes, and HIV, the virome remains far less studied. [1].

Just as HIV infection disrupts the bacterial microbiota, it is reasonable to assume that it also affects the human virome. While the bacteriome has been widely investigated in individuals with HIV infection, the role of the virome in HIV-related pathogenesis remains considerably less understood.

In people living with HIV (PLWH), gut microbiota composition is significantly altered compared to the general population, with reduced diversity, barrier disruption, and chronic local and systemic inflammation driven by microbial translocation and sustained immune activation, all of which contribute to immunopathogenesis and disease progression [2,3,4,5]. In PLWH, gut dysbiosis has been linked to immune activation, microbial translocation, and differential responses to antiretroviral therapy (ART), underscoring reciprocal interactions between microbial imbalance and host immunity [2]. These alterations originate early, as HIV replication within the Gut-Associated Lymphoid Tissue (GALT) depletes CD4^+^ T-cell subsets, weakens the epithelial barrier, and promotes systemic immune activation [3,6,7]. Bacterial and viral dysbiosis may synergize in sustaining this cycle, with virome shifts not only exacerbating bacterial alterations but also exerting direct effects on mucosal and systemic immunity (e.g., certain viral populations have even been associated with immune restoration under ART, highlighting the complex and bidirectional role of the virome in HIV pathogenesis) [3]. Collectively, these findings support a model where bacteriome and virome alterations converge to perpetuate barrier dysfunction, chronic inflammation, and HIV-associated comorbidities [8,9].

In recent years, the life expectancy of PLWH has risen considerably, transforming HIV infection into a chronic and manageable condition in the era of ART [10,11]. While the broad implementation of ART has been pivotal in lowering HIV-related morbidity and mortality, long-term therapy may have significant, yet not fully understood, effects [12]. Investigating how ART influences the plasma virome and whether commensal viruses contribute to persistent immune activation and inflammation is, therefore, essential [13]. Given the critical role of the human virome in HIV pathogenesis and its associated complications, and considering the intricate interplay between infection, ART, and the virome, this review aims to provide an updated summary of current knowledge, with a particular focus on the commensal virome.

## 2. Composition of the Human Virome

The human virome consists of bacteriophages, which infect bacteria; viruses targeting other cellular microorganisms, such as archaea; viruses that infect human cells; and transient viruses acquired from dietary sources, including plant-derived viruses. Moreover, virome diversity and composition change across life stages. Neonates exhibit high phage and low bacterial diversity; as the gut microbiota develops, bacterial diversity increases while phage diversity declines. In healthy adults, a stable bacterial–phage homeostasis is maintained, whereas aging is associated with a decline in both viral and bacterial diversity [14,15].

Currently, within the human virome, the intestinal component is the most extensively studied [16]. Although the majority of the human virome is believed to consist of bacteriophages, viruses that infect human cells still constitute an important component [17]. While some viruses are responsible for acute infections and others establish long-term latency, a subset has been suggested to engage in benign colonization without a clear link to disease, acting as a putative persistent ‘passengers’ or ‘commensals’ [17]. Sequencing studies of the virome have revealed several novel lineages of human viruses that appear to behave as commensals [18]. One example is the *Anelloviridae* family, comprising non-enveloped, single-stranded DNA viruses with small circular genomes—such as torque teno virus, torque teno mini virus, and torque teno midi virus [19]. These viruses are highly diverse and can be found across many human body sites, although, to date, they have not been linked to any specific pathogenic effects [20]. Greater abundance of *Anelloviridae* has been found in immunocompromised individuals, including the recipients of transplants, PLWH and those on immunosuppressive medications, indicating that members of the family *Anelloviridae* are normally under host immune control [21,22]. *Redondoviridae*, a recently identified family of small circular DNA viruses, also appears to be widespread commensals, predominantly inhabiting the respiratory tract [23].

High inter-individual variability in the human virome, commonly referred to as beta diversity (differences in viral community composition between individuals), has been consistently reported. In contrast, the virome of a healthy adult remains relatively stable over time within the same individual. Alpha diversity (the diversity within a single community) varies across individuals but is generally maintained over time in healthy adults. Perturbations of this intra-individual temporal stability or reductions in alpha diversity are often associated with disease [17].

Alterations in the enteric human virome and bacterial microbiome have been associated with inflammatory bowel disease (IBD), obesity, and modifications in host behavior [24]. Enteric eukaryotic viruses can cause conditions such as gastroenteritis, enteritis, or colitis, directly impacting human health by causing [25]. Bacteriophages, on the other hand, may influence gut health indirectly by modulating bacterial communities and can also interact directly with the human immune system [26]. In IBD, an inverse relationship has been observed between bacterial microbiome diversity and bacteriophage richness, indicating a potential antagonistic interaction between bacteria and bacteriophages during intestinal inflammation [24].

## 3. Virome of Different Human Body Sites

Recent research has mapped the human virome across various body sites, uncovering diverse viral populations throughout [17]. Bacteriophages are widely distributed, yet their composition varies between anatomical locations—such as the saliva, gastrointestinal tract, and respiratory tract—reflecting differences in the resident bacterial hosts [27].

- Gut virome. The gastrointestinal tract is typically the site with the highest viral abundance, exhibiting considerable temporal stability within individuals but substantial variability between different people [28,29]. Analysis of virome sequencing from fecal samples indicates that bacteriophages constitute the majority of identifiable viral populations, accounting for over 90%, with most of them belonging to the order *Caudovirales* (*Podoviridae* and *Siphoviridae*) (dsDNA viruses) along with the spherical *Microviridae* (ssDNA viruses). Gut virome comprises also eukaryotic RNA viruses (e.g., rotaviruses, coronaviruses, sapoviruses and plant viruses) and eukaryotic DNA viruses (e.g., herpesviruses, adenoviruses and anelloviruses) [17].
- Blood virome. Studies conducted on the plasma virome show instead the predominance of eukaryotic viruses such as *Anelloviridae*, *Herpesviridae* and *Picornaviridae* and low abundance of phages (most prevalent were *Caudovirales* and *Microviridae* [30,31]. Some investigations, however, have reported higher levels of phage DNA in the blood of patients with cardiovascular disease and HIV compared to healthy individuals [29].
- Respiratory tract virome. Virome analyses of respiratory samples—including sputum, nasopharyngeal swabs, and bronchoalveolar lavage—indicate that the healthy human lung and respiratory tract can harbor extensive viral communities [32,33]. Research indicates that *Anelloviridae*, *Redondaviridae*, and *Herpesviridae* are the most prevalent viruses in samples from the human respiratory tract [34]. Bacteriophages detected in the lungs largely originate from the abundant bacterial communities of the mouth and upper respiratory tract, and their composition resembles that observed in the gut [17].
- Breast milk virome. Knowledge of the breast milk virome remains limited. In healthy U.S. women, most viruses detected in breast milk were bacteriophages belonging to the *Myoviridae*, *Siphoviridae*, and *Podoviridae* families, with eukaryotic viruses being rare [35]. However, only a small number of pathogenic viruses, such as HIV, cytomegalovirus (CMV), and human T-lymphotropic virus type 1 (HTLV-1), are known to be transmitted via breast milk [36]. These and other viral components may influence the infant gut microbiome and virome through immune modulation and inflammatory effects, with potential consequences for child health. Studies from Italy and the United States provide evidence for vertical transmission of the virome, as bacteriophage compositions in breast milk and infant stool from mother–infant pairs show significant similarity [37]. Consequently, changes in the breast milk virome could influence the initial development of both the infant virome and bacterial microbiome, potentially affecting long-term health outcomes [38].
- Other sites viromes. Little information is available on virome populations in other sites such as the nervous system, skin or urogenital system [39]. Anyway, even body sites largely isolated from typical microbial colonization, such as cerebrospinal fluid (CSF), exhibit low levels of viruses, including bacteriophages [17]. *Siphoviridae* and *Myoviridae* have been reported as predominant viral families in the CSF [40].

The extent to which the local virome reflects true viral replication, systemic viral circulation, or technical artifacts such as reagent contamination has yet to be fully determined [17].

The viral families of the human virome in different body sites are detailed in Table 1.

Recently, it has been suggested that local viromes can influence distant organs [41]. For instance, behavior and cognitive performance seem to be influenced by gut phages (*Siphoviridae* and *Microviridae*) [42].

## 4. Approaches to Investigating the Virome and Its ‘Dark Matter’

In contrast to bacteria, which can be analyzed using universal 16S rRNA primers present in all bacterial genomes, viruses lack conserved genes that would allow for a universal detection approach [43].

The first step in a virome study typically involves isolating virus-like particles (VLPs) from the samples of interest via filtration, which prevents the unnecessary sequencing of non-viral nucleic acids [17]. Traditional viral detection relies on culturing viruses in susceptible cells and identifying viral signatures through various methods. Molecular approaches, such as polymerase chain reaction (PCR), are widely employed in virology laboratories for diagnosing viral infections [44]. Other common techniques for detecting viral antigens include enzyme-linked immunosorbent assay (ELISA), Western blotting, and immunofluorescence [43]. For metagenomic analyses of complex samples, high-throughput methods like microarrays are often used [45]; however, these approaches require bioinformatic analysis of conserved regions across diverse viral antigens and are limited to detecting known viruses [43].

The so-called ‘viral dark matter’ refers to sequences that show no similarity to entries in existing databases, representing a major challenge for virologists and bioinformaticians. Approaches to tackle this issue include mining publicly available viral datasets, employing untargeted metagenomic strategies, and applying advanced bioinformatic tools to identify, quantify, and classify viral species [46].

Shotgun metagenomics has allowed the study of the human microbiome without the need for cultivation. Given the significant challenges in experimentally isolating viruses, metagenomic sequencing is essential for comprehensively cataloging viral diversity [47]. Despite providing an unprecedented depth of microbiome profiling, metagenomic identification of viruses remains challenging. Key limitations include the immense diversity of viruses and the absence of universal genetic markers, which complicate the extraction of viral sequences from raw data [47]. To address these issues, numerous computational tools have been developed for viral detection in metagenomes, some of which are capable of de novo viral prediction [48]. However, all existing approaches still rely, at least in part, on previously characterized viral genomes, whether through direct sequence similarity, recognition of viral genomic signatures, or machine learning models trained to distinguish viral sequences [47].

## 5. HIV-Driven Alterations in the Human Virome

HIV can influence the human virome through multiple mechanisms. First, HIV-associated immune dysfunction reduces the host’s ability to control viral replication, favoring expansion of eukaryotic viruses and shifts in phage populations [49]. Second, HIV-driven damage to mucosal barriers—especially in the gut—facilitates translocation of viral particles and promotes systemic dissemination of enteric viruses [3]. Third, chronic inflammation and altered cytokine signaling reshape the ecological balance between bacteria and bacteriophages, indirectly modulating phage–bacteria dynamics [3]. Finally, ART itself can influence virome composition by restoring immune surveillance or modifying microbial communities [13].

It is now well established that the microbiome profiles of healthy individuals differ markedly from those of PLWH, and those differences have been described in several body compartments, but most information is focused on the gut microbiome and its impact on comorbidities [3].

Considerable progress has been made in understanding the relationships and mechanisms linking the bacteriome and HIV in humans; however, much of the research on the virome and its association with HIV/SIV has been conducted in primates [1]. In gorillas, SIVgor infection was associated with significant increases in *Herpesviridae* and *Reoviridae*, while *Rhabdoviridae* levels were higher in uninfected individuals [50]. Pathogenic SIV infection of rhesus monkeys also showed an expansion of the enteric virome, including increases in parvoviruses and picornaviruses as well as adenoviruses, which were associated with enteritis and may play a role in AIDS enteropathy [51].

Expansion of the enteric virome during SIV infection in primates can be mitigated through appropriate vaccination, potentially reducing the incidence of AIDS-associated enteropathy [52]. Viruses detected in primate feces have also been shown to infect other tissues and enter systemic circulation [51], likely a consequence of the compromised intestinal epithelium resulting from chronic SIV infection [1]. The altered enteric virome may reflect the host’s diminished capacity to control viral populations that would normally be contained, due to impaired gut immunity [1].

In the acute phase of infection, *Anelloviridae* abundance in blood was positively correlated with CD4^+^ T cell counts and negatively correlated with SIV viral load, whereas *Parvoviridae* and *Circoviridae* exhibited inverse relationships with these disease markers [53,54].

Few data are focused on the virome of PLWH in several body sites:

- Gut microbiome. The human gut ecosystem is shaped by the interaction among the bacteriome, virome, and phageome, which are in constant dialogue with the host. During HIV infection, immune alterations and bacterial dysbiosis disrupt this communication [3]. Phages regulate bacterial homeostasis through lytic, lysogenic, or pseudo-lysogenic cycles, and their overwhelming abundance positions the virome as a key modulator of the microbiota [49].

Bacteriophages represent a large fraction of the gut virome, and the microbiome composition (beta diversity) is altered in PLWH compared to healthy individuals.

In PLWH, the depletion of beneficial intestinal bacteria (e.g., short-chain fatty acid producers) may be linked to an increased abundance of phages targeting these taxa, whereas the predominance of pro-inflammatory species such as *Prevotella* spp. appears to coincide with a reduction in their phage predators, thereby favoring bacterial expansion. A notable example is the *Bacteroides* phage B40-8, which infects *Bacteroides fragilis*: in PLWH, enhanced activity of this phage may further contribute to the depletion of protective *Bacteroides* spp. populations.

In treatment-naive PLWH, an increase in *Caudoviricetes* (dsDNA) phages and a decrease in *Malgrandaviricetes* have been observed in the gut [3]. Notably, *Caudoviricetes* are also elevated in inflammatory bowel disease (IBD), suggesting a potential association with heightened inflammation and increased gut permeability [55].

Multiple studies have reported that HIV infection in humans can lead to an increase in enteric adenoviruses, while the composition of the bacteriophage population remains largely unchanged [51,53].

A study by Villoslada et al. highlights the importance of analyzing both DNA and RNA viruses in the gut virome. Unlike other reports, this study found that plant- and fungi-infecting viruses—primarily members of the *Virgaviridae*—were the most abundant, followed by small circular viruses and animal-infecting viruses, indicating that the majority of eukaryotic viruses in the gut microbiome are likely derived from the diet [55].

- Blood virome. Previous cross-sectional studies have demonstrated that HIV infection alters the plasma virome, primarily through the expansion of eukaryotic viruses [43]. While virome expansion associated with HIV occurs in both blood and gut, the specific eukaryotic viruses involved differ substantially, reflecting the distinct virome profiles across various tissues and organs [56]. Different from the main contribution of adenovirus to the gut virome expansion, plasma virome expansion caused by HIV infection is mainly driven by anelloviruses, which dominate the plasma virome [57].
- Genital Tract virome. Several studies also report high prevalence of *Papillomaviridae* in the oral and genital mucosa of individuals with severe immunodeficiency, and they are strongly implicated in the development of neoplastic lesions [1,58]. Recently, it has been described that the cervicovaginal virome of women living with HIV changes during ART, with anelloviruses abundance reduction during ART and concomitant with CD4^+^ increase [59].
- Respiratory tract virome. To date, published data on the lung virome in the context of HIV infection remain very limited. In a study involving a small cohort of PLWH, bronchoalveolar lavage (BAL) primarily revealed the presence of anelloviruses, followed by bacteriophages. Additionally, Epstein–Barr virus (EBV), *Retroviridae*, and *Parvoviridae* were detected, with evidence of active replication in some cases, indicating ongoing viral activity [60].

**Figure 1:**
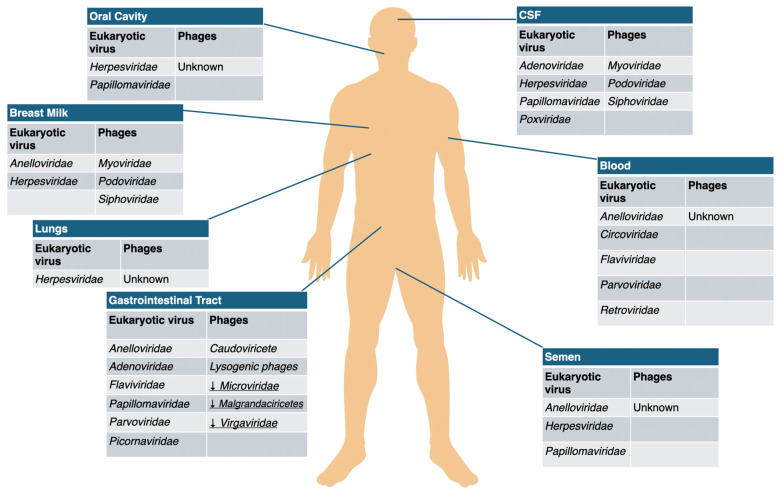
Overview of eukaryotic viral families and bacteriophage taxa identified in various anatomical compartments of HIV-naive individuals. Arrows indicate taxa that are increased or decreased compared with control groups.

Distribution of eukaryotic viruses and phage populations in multiple body sites among HIV-naïve individuals is fully represented in Figure 1.

## 6. The Virome in the Era of ART

The advent of ART has dramatically improved the prognosis of PLWH, extending life expectancy and enhancing quality of life by suppressing viral replication and enabling immune reconstitution. Nevertheless, its impact on the human virome remains limited: while ART improves immune status and reduces HIV replication, it only partially restores virome balance and does not fully reverse its dysregulation. For instance, in a longitudinal study on SIV-infected macaques, delayed ART initiation eliminated SIV replication, but ART was unable to normalize the expansion of *Anelloviridae* and *Parvoviridae* associated with the infection, which continued to stimulate pro-inflammatory cytokines [54]. Similarly, in PLWH, ART strongly suppresses HIV replication, but it only partially improves virome composition in terms of diversity, with persistent anomalies even after years of treatment [53,55]. Effective ART reduces HIV viremia, but it seems to only modestly reduce the prevalence and abundance of anelloviruses without normalizing HIV-induced cytokine and inflammatory alterations, with persistently elevated levels of cytokines (e.g., IL-10, GM-CSF, VEGF) even after 12 months of treatment [13,61].

Villoslada-Blanco et al. [55] have described that ART can increase the abundance of intestinal bacteriophages and reduce the expansion of certain viral families such as *Anelloviridae*, but it does not fully correct the increase in lysogenic phages and the reduction in phages that infect Proteobacteria, nor does it normalize viral diversity to the levels of HIV-negative individuals [3,62].

Virome changes under ART appear to evolve gradually. Prospective studies suggest that short-term ART (<1 year) fails to normalize the expansion of commensal plasma viruses such as *Anelloviridae* and pegivirus, a member of the *Flaviviridae* family, which remain elevated and associated with altered cytokine profiles [13]. Partial normalization is observed only after long-term ART (>2 years), with a reduction in human viral abundance and increased detection of plant viruses belonging to the *Tobamovirus* genus, indicative of enhanced immune system reconstitution [63].

The distinction between short and long-term is therefore crucial: virome alterations persist despite HIV suppression in the short-term, while long-term ART drives partial but incomplete recovery with persistent imbalance likely contributing to chronic inflammation and comorbidities [13,53,55].

ART also has measurable effects on virome diversity. In a Spanish cohort, ART partially restored the alpha diversity of bacteriophages, bringing it closer to that observed in HIV-negative controls, whereas beta diversity remained at an intermediate level between HIV-naive and HIV-negative individuals [55]. Similarly, CSF analysis from PLWH on ART showed reduced alpha diversity compared to HIV-negative controls, despite a broadly similar composition in immune-reconstituted individuals [42]. These findings suggest that while ART partially mitigates diversity loss, it does not fully restore virome balance.

**Figure 2:**
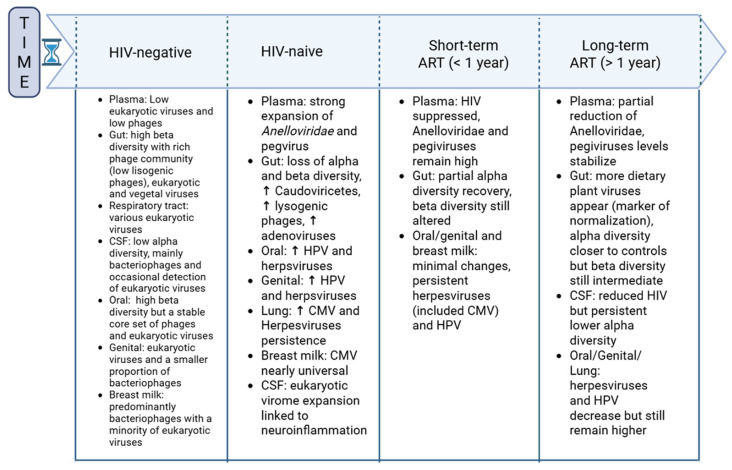
Changes in virome composition in HIV-negative individuals, PLWH before and after ART initiation. Viral types and virome variation are summarized from 1, 3, 13, 17, 27–34, 40, 43, and 51–66. Abbreviations: ART, antiretroviral therapy; CSF, cerebrospinal fluid; HPV, human papillomavirus; CMV, cytomegalovirus; ↑ expansion. Created in https://BioRender.com.

Changes in virome composition in PLWH before and after ART initiation are fully explained in Figure 2.

It has been recently described that ART effects on the human virome may vary depending on the regimen. Integrase inhibitors (INSTIs) appear to exert a more favorable impact on the intestinal virome than other classes of antiretrovirals, allowing a partial recovery of phage diversity [55]. Nonetheless, the persistence of imbalances even under INSTI-based therapy suggests that no regimen can fully re-establish viromic homeostasis.

An additional possibility is that ART itself contributes to shaping the human virome in PLWH. This influence may explain why virome normalization after ART initiation remains incomplete, with certain viral and bacteriophage populations not fully restored to levels observed in HIV-negative individuals. Evidence from both animal and human studies indicates that ART—particularly nucleoside reverse transcriptase inhibitor (NRTI)-based regimens—can induce specific alterations in gut microbial diversity and composition, leading to reduced alpha diversity and shifts in bacterial and viral genera, regardless of HIV infection itself [64,65,66]. These ART-induced alterations further compound the dysbiosis initially caused by HIV infection, thereby contributing to the persistence of viromic and microbiomic perturbations despite effective virological suppression.

## 7. Immunological and Clinical Implications

Alterations in the human virome during HIV infection seem to be closely associated with impaired immune responses, chronic inflammation, and the development of comorbidities.

**Figure 3:**
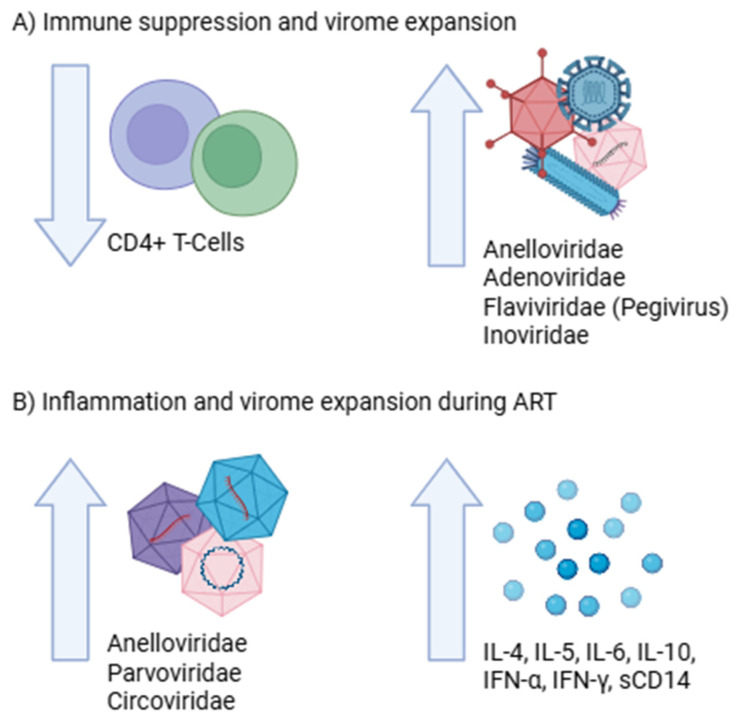
Immune perturbation and virome expansion in HIV and ART contexts. (**A**). As immunodeficiency progresses and CD4^+^ T cell counts decline, these viral taxa expand. (**B**). The expansion of these taxa contributes to chronic inflammation by promoting cytokine production. Created in https://BioRender.com.

In terms of immune function, expansion of *Anelloviridae*, *Adenoviridae*, and *Inoviridae* bacteriophages has been associated with low CD4^+^ T cell counts and more severe immunodeficiency (Figure 3A). The simultaneous decrease in *Anelloviridae* abundance and CD4^+^ T cell count during the early acute phase may reflect the loss of CD4^+^ T cells, which serve as target cells for anelloviruses [54,63]. In vitro studies have shown that certain human anellovirus isolates can stimulate the secretion of pro-inflammatory cytokines, including IL-6, IL-10, and IFN-γ, via the TLR-9 pathway, sustaining systemic inflammation even after ART initiation [67]. Similarly, positive correlations have been observed between *Parvoviridae* and *Circoviridae* abundances and pro-inflammatory cytokines such as IL-4, IL-15, IL-6, and TNF-α during the chronic phase and under ART, suggesting that these viruses may also contribute to persistent inflammation (Figure 3B) [54].

Several studies have proposed anelloviruses as biomarkers of immune reconstitution: high plasma levels before ART initiation predict poor immune recovery with persistently low CD4^+^ counts during follow-up [63,68]. In PLWH, Torque teno virus (TTV) DNA levels have been found to inversely correlate with CD4^+^ T cell counts, and in children and young adults with vertically acquired HIV, TTV has been associated with altered CD4/CD8 ratios and persistent inflammatory markers, suggesting a potential prognostic role in both children and adults [69,70,71,72,73]. In the intestinal tract, the presence of *Anelloviridae* has also been identified as a marker of immunodeficiency, correlating with low CD4^+^ levels and poor therapeutic response [63]. Although ART gradually reduces anellovirus levels, high loads may persist in individuals with incomplete immune reconstitution [68].

Other viruses can reflect the response to ART. For example, increased pegivirus levels during ART have been associated with slower lymphocyte reconstitution [13].

However, pegivirus has also been linked to beneficial outcomes. Several studies reported that coinfection with pegivirus can inhibit HIV-1 replication and improve patient survival [68,74,75]. Unlike anellovirus, pegivirus abundance is lowest in AIDS patients, and its shedding shows an inverse association with host immune status [68].

Together, these findings suggest that pegivirus coinfection may have complex and context-dependent effects: while it may slow lymphocyte reconstitution during ART, it can also exert protective effects by suppressing HIV-1 replication and enhancing survival. Anelloviruses and pegiviruses are the most abundant components of the blood virome, and their inverse correlation suggests distinct immunomodulatory roles. Persistent infection with these viruses may therefore serve as potential indicators of HIV-1 disease progression [68].

Within the gut, the expansion of *Adenoviridae* and *Anelloviridae* correlates with enteropathy, microbial translocation, and elevated levels of sCD14, all of which contribute to heightened immune activation [3,53]. The loss of bacteriophage diversity and depletion of protective phages (e.g., *Inoviridae*) further exacerbate bacterial dysbiosis, favoring the expansion of pro-inflammatory taxa such as Enterobacteriaceae. Even after ART initiation, residual viral dysbiosis contributes to persistent immune activation and cytokine dysregulation, with elevated levels of IL-10, GM-CSF, and VEGF despite virological suppression and restored CD4^+^ counts [61,76]. This unbalanced state facilitates microbial translocation and chronic immune activation, promoting inflammatory and metabolic comorbidities such as metabolic syndrome and cardiovascular disease [3,53,55,77].

In the central nervous system, the presence of virome in CSF has been associated with biomarkers of neuroinflammation and impaired cognitive performance, independent of HIV viremia [42]. Specifically, bacteriophages, including *Siphoviridae*, *Myoviridae*, and *Podoviridae,* are correlated with levels of S100β and β-amyloid42, while human viruses, such as *Herpesviridae*, *Papillomaviridae*, and *Adenoviridae*, are linked to impaired cognitive function. In contrast, plant viruses are associated with low β-amyloid42 levels and improved cognitive performance. These findings suggest that the virome may influence brain health and serve as a potential marker of neuroinflammation, with implications for the early detection and prevention of neurocognitive disorders.

## 8. Future Perspectives and Conclusions

Virome composition can be characterized as the prevalence (proportion of hosts infected), abundance (viral load), and distribution (temporally or spatially) of viruses within individuals. However, our understanding of the complex viral networks within the human microbiome and their impact on health and disease remains limited.

The field of virome research is undergoing a transition from descriptive studies to hypothesis-driven approaches aimed at uncovering the underlying mechanisms and biological consequences of virome diversity.

Despite the progress of ART, current evidence shows that the human virome does not recover a full physiological state in PLWH. Persistent imbalances, particularly involving *Anelloviridae*, reflect incomplete immune reconstitution and contribute to maintained chronic inflammation.

Studies investigating the human virome in HIV infection remain relatively limited, frequently characterized by small sample sizes and a predominantly cross-sectional design. There is a lack of comprehensive longitudinal trials and comparative studies between different ART regimens. Furthermore, most of the analysis focuses on viral DNA, thereby overlooking RNA viruses and potentially underestimating the true complexity of the virome [51].

Emerging approaches, including deep metagenomics sequencing and integrated multi-omic analysis, may facilitate a better understanding of the functional role of the human virome, discerning between commensal viruses, opportunistic and pathogens. The identification of viromic markers, such as anelloviruses, could be useful in clinical practice as a tool for the follow-up of the immune function.

It will be essential to conduct longitudinal studies to understand the evolution of the virome from the moment of acute infection through years of ART, including geographically diverse populations, women, children, and people with metabolic or neoplastic comorbidities [3].

In summary, HIV and ART profoundly reshape the human virome, with implications for persistent inflammation, susceptibility to co-infections, and immunological recovery. The virome emerges not only as a marker of immune dysfunction but also as an active player in modulating clinical outcomes. Understanding and modulating these interactions represents a new development in HIV management.

## 9. Search Strategy and Selection Criteria

A search was conducted using MEDLINE and Google Scholar to identify peer-reviewed, English-language studies published up to 1 November 2025, examining the composition, diversity, and dynamics of the human virome in health, HIV infection, and during ART. Relevant references were identified through a combination of search terms, including: “human virome,” “microbiome,” “HIV,” “antiretroviral therapy,” “bacteriophage,” “Anelloviridae,” “virome diversity,” and “immune activation.”

Studies were selected through a two-step process comprising an initial screening of titles and abstracts followed by a detailed full-text review of shortlisted articles. This selection process was independently undertaken by two authors, with disagreements resolved through discussion and consensus.

It is important to note that this work is not a systematic review. Instead, the final selection of references was curated based on multiple criteria, including publication date, originality, accessibility, and relevance to the scope of this narrative review.

## Figures and Tables

**Table 1:** Virus families found in different body sites in the general population [17,27,28,29,30,31,32,33,34,40].

Body Site	Eukaryotic Viruses	Phages
Blood	*Anelloviridae*	*Inoviridae*
	*Herpesviridae*	*Microviridae*
	*Picornaviridae*	*Myoviridae*
		*Podoviridae*
		*Siphoviridae*
Skin	*Adenoviridae*	*Myoviridae*
	*Anelloviridae*	*Podoviridae*
	*Circoviridae*	*Siphoviridae*
	*Herpesviridae*	
	*Papillomaviridae*	
	*Polyomaviridae*	
Nervous System	*Herpesviridae*	*Myoviridae*
		*Podoviridae*
		*Siphoviridae*
Oral Cavity	*Anelloviridae*	*Myoviridae*
	*Herpesviridae*	*Podoviridae*
	*Papillomaviridae*	*Siphoviridae*
	*Redondoviridae*	
Lung	*Adenoviridae*	*Inoviridae*
	*Anelloviridae*	*Microviridae*
	*Herpesviridae*	*Myoviridae*
	*Papillomaviridae*	*Podoviridae*
	*Redondoviridae*	*Siphoviridae*
Gastrointestinal Tract	*Adenoviridae*	*Inoviridae*
	*Anelloviridae*	*Microviridae*
	*Caliciviridae*	*Myoviridae*
	*Circoviridae*	*Podoviridae*
	*Herpesviridae*	*Siphoviridae*
	*Picornaviridae*	
	*Virgaviridae*	
Urinary System	*Papillomaviridae*	*Myoviridae*
	*Polyomaviridae*	*Podoviridae*
	*Herpesviridae*	*Siphoviridae*
Vagina	*Anelloviridae*	*Microviridae*
	*Herpesviridae*	*Myoviridae*
		*Podoviridae*
		*Siphoviridae*
Semen	*Anelloviridae*	Unknown
	*Herpesviridae*	
	*Papillomaviridae*	

## Data Availability

No new data were created or analyzed in this study. Data sharing is not applicable to this article.
